# Production and Properties of Starch Citrates—Current Research

**DOI:** 10.3390/foods9091311

**Published:** 2020-09-18

**Authors:** Antoni Golachowski, Wioletta Drożdż, Magdalena Golachowska, Małgorzata Kapelko-Żeberska, Bartosz Raszewski

**Affiliations:** 1 Department of Food Storage and Technology, Faculty of Biotechnology and Food Sciences, Wrocław University of Environmental and Life Sciences, C.K. Norwida 25, 50-375 Wroclaw, Poland; antoni.golachowski@upwr.edu.pl (A.G.); malgorzata.kapelko@upwr.edu.pl (M.K.-Ż.); bartosz.raszewski@upwr.edu.pl (B.R.); 2 Institute of Health Sciences, Collegium Medicum, University of Opole, ul. Kopernika 11a, 45-040 Opole, Poland; m.golachowska@gmail.com

**Keywords:** starch, methods of starch citrification, the properties of starch citrate, resistant citrate starch

## Abstract

Starch modification by chemical reaction is widely used to improve the properties of native starch. Modified by citric acid, starch is characterized by specific properties resulting from the presence of citrate residues and as a result of cross-linking starch. The chemicals used for preparing starch citrates are safe for human health and the natural environment compared to the harsh chemicals used for conventional modifications. Starch citrates are traditionally produced by heating starch–citric acid mixtures in semi-dry conditions or by a heat moisture treatment. The conditions of the modification process (roasting temperature, heating time, citric acid dose) and the botanic source or genotype of starch determine the degree of substitution and the properties of the obtained preparations. Changes of starch properties occurring during esterification lead to reduced relative crystallinity, resulting in a decrease in the affinity for water, the gelatinization parameters, and the viscosity of starch citrate. However, one of the most important outcome of the modification is the formation of resistant starch (RS), which has increased resistance to the action of amylolytic enzymes. Currently, new methods for producing starch citrates with improved functional and rheological properties while maintaining the highest possible content of resistant starch are being sought. The article presents an overview of recent studies on the production, properties. And applicability of starch citrates with special attention paid to their role as preparations of resistant starch (RS). The use of citric acid for modification of starch is better for the technology process, while using cross-linking is better than simply using esterification.

## 1. Introduction

Starch is one of the most ubiquitous storage substances in nature. In its native form, starch has limited applications due to properties that are not always desirable for certain types of processing. Examples of these shortcomings include its inability to withstand the high temperature, pressure, and some strong chemical reagents use in most industrial food and pharmaceutical processes. Starch modification, which implies the alteration of the physical and chemical characteristics of native starch, is used to improve its properties. The chemical modification of starch is directly related to the reactions of the hydroxyl groups of the starch polymer. Almost all glucose residues in starch chains possess three free-hydroxyl groups capable of oxidation, etherification, and esterification. Starch modification through esterification involves starch chain reactions with inorganic and organic acids, salts of inorganic acids, and anhydrides or chlorides of organic acids. A commonly used modified starch in the food industry is acetylated distarch adipate, obtained by the esterification of starch with adipic acid and acetic anhydride [1]. This preparation is characterized by a higher thermal stability, shear resistance, and acid resistance compared to native starch and forms strong and stable gels that are resistant to retrogradation [2]. For this reason, these starch modifications are widely used in food products as a thickener, stabilizer, and binder [3]. However, the acetylated distarch adipate has applications that are regulated by the degree of substitution (DS) or the percentage of acetyl groups (% Ac). These characteristics determine the use of starch acetate, for example, for food application; the United States Food and Drug Administration (FDA) recommends a percentage of acetyl groups of less than 2.5 g/100 g [4].

Recently, scientists have focused a lot of attention on the preparation and testing of the properties of starch modified with citric acid. Citric acid (2-hydroxypropane-1,2,3 tricarboxylic acid, C_6_H_8_O_7_) itself is recognized as a safe food additive (GRAS) and is used widely in the food, cosmetic, and pharmaceutical industries [5]. The use of citric acid is considered safe for both the natural environment and human health [6]. The JECFA (Joint FAO/WHO Expert Committee on Food Additives) monograph on modified starches does not set any particular limitations on the use of starch citrate [7]. Citric acid has been used for starch modifications since the mid-20th century. A method for the production of its starch esters was patented in 1949 [8], whereas a preparation of starch crosslinked with this acid was patented in 1960 [9]. Citric acid is relatively often used in current investigations addressing starch modifications [6,10,11,12,13,14,15,16,17]. Mainly it serves as an acidity regulator. Citric acid is widely used as a reagent in reactions of esterification and crosslinking of starch conducted under various conditions and types of starch. It is also used for starch treatment at temperatures lower than its pasting temperatures [18,19,20,21,22,23] and in the heat moisture treatment of starch [24,25]. In the polymeric materials produced based on starch, it is used as a compatibilizer [26], a plasticizer [9], and a crosslinking agent [11,12,13,14,27,28]. In addition, it serves the role of a catalyst in carbonspheres production from starch that are used, i.e., lithium batteries [15]. In the production of starch citrate, citric acid is converted to its anhydride, which forms ester bonds with the hydroxyl groups in starch [29]. However, the superiority of citrates over other modifications can be explained by the cross-linking process itself. Crosslinking is a type of chemical modification that offers greater stabilization of the starch structure. It can be performed with polycarboxyl organic acids, which form bridges by reacting with hydroxyl groups of anhydroglucoses from the neighboring chains, thereby stabilizing the polymeric backbone. Citric acid was chosen for this overview because its heating with starch ensures high crosslinking of the latter. Its additional advantage to starch modification is the possibility of modelling starch resistance to amylolysis in a whole variability range (0–100%) because it is correlated with the process temperature and acid dose [30].

This work aims to present an overview of the current investigations on the properties of starch preparations produced via esterification/crosslinking with citric acid, with special attention paid to their role as preparations of resistant starch (RS). Furthermore, the specification, variant methods, and changes in the variety, properties, and processing of starch citrate are summarized in this article.

## 2. The Methods of Starch Citrate Preparation

In recent studies, processes of esterification/crosslinking with citric acid have been conducted with various starch types, including both these produced on the large industrial scale, i.e., “normal” and waxy maize starch [27,31,32,33,34,35,36], wheat starch and its A and B fractions [22,37], potato starch [38,39,40,41,42,43], rice starch [44,45], and cassava starch [14,43,46,47], as well as with starch extracted from local plants, i.e., from banana [43,48], taro [49], yam [50,51], sweet potato [43,52], acha and iburu [53], kidney bean [54], lentil [43], and barley [16]. Investigations have been carried out with native starch (non-modified) and with starch modified using physical and chemical methods—porous starch [11], retrograded starch [41,42], starch nanocrystals [11], carboxymethyl starch [55], starch coated with nanoparticles [56], and alkaline starch suspension [57].

The process of starch esterification has been performed with various methods, which affected the degree of substitution and properties of the modified preparations (Table 1). The most frequent has been the “dry” esterification/crosslinking described by Klaushofer et al. [7], in which starch is first mixed with an aqueous solution of citric acid and then dried and roasted. The preparation obtained is then rinsed with water or/and an ethanol solution, dried again, and disintegrated.

This method has been modified by many researchers and these modifications concerned the use of various citric acid to starch ratios (from 1 g to 60 g of citric acid per 100 g of starch) and performing this reaction at various pH values (3.5–5.5), roasting temperatures (110–140 °C), and roasting times (1–5 h).

Different conditions producrf modified preparations with a substitution degree ranging from 0.01 to 0.42, which was found to depend on the type of starch material and its earlier modifications [37,41,42,43], citric-acid-to-starch ratio [11,37,38,39,41,42,43,46,47,52,58], pH value of the reaction [35,58], and the time and the temperature of roasting [35,42,47].

“Dried” esterification was carried out not only in roasters but also in extruders. A study conducted by indicates that the molar substitution degree of the starch citrates obtained in the process of reactive extrusion ranges from 0.01 to 0.03 depending on the amount of citric acid, the temperature of extrusion, and the number of passages. The effect of the amount of citric acid added to extruded starch on the substitution degree of the modified preparation was also observed in the work by Ye et al. [45]. The degree of substitution and the properties of starch citrates produced with the extrusion method were also affected by the addition of galactomannans (guar gum, tara gum, and locust bean gum) [31].

Another procedure of “dry” esterification was described by Kim and Min [36]. The reaction was induced by microwave-discharged cold plasma. The molecular degree of substitution of the modified preparation reached 0.012–0.015 and was similar to that of starch modified with the “classic” method, i.e., through roasting at a temperature of 135 °C for 1 h.

Starch esterification and crosslinking may also be induced by exposing the reaction mixture to ultraviolet irradiation and applying sodium benzoate as a photosensitizer. This method was used for film production from thermoplastic starch [10].

The method of “dry” esterification of the starch through its roasting with citric acid was also used to produce many modified preparations of starch intended not for the food industry but for pharmaceutical or technical purposes. Preparations of this type included crosslinked starch nanocrystals potentially used as drug delivery vehicles and reinforcements for nanocomposites with a hydrophobic polymer matrix [28], crosslinked porous starch used for the absorption of heavy metal ions [11], starch films with good barrier and mechanical properties [13,28], novel starch foams that may replace petroleum-based foams [12], or hydrogels ensuring appropriate release of active substances of a drug [57].

Apart from the above-discussed “dry” esterification method described in a few works [49,50,52,53], recent investigations on starch citrates have also addressed “wet” esterification performed according to Agboola et al. [59]. This method consists in the localization of an aqueous starch suspension, the addition of a solution of citric acid and a solution of sulfuric acid as a catalyst, and keeping this suspension at room temperature. The next steps include rinsing with water to remove residues of reagents, drying, and disintegration. Depending on the amount of citric acid, the degree of substitution of the modified preparations ranged from 0.002 to 0.023 [59]. The “wet” esterification conducted under slightly different conditions was also employed to produce hydrogels capable of encapsulating nutraceuticals [57].

Hernandez-Jaimes et al. demonstrated the feasibility of producing starch citrates in the aqueous medium with the electrochemical method using a triangular-shaped potential cycle, with substitution degree of the obtained preparations ranging from 0.02 to 0.12 depending on the number of cycles [33]. The esterification/crosslinking of starch in an aqueous suspension was also used to produce “non-food” preparations, e.g., during production of curcumin-loaded starch coated with iron oxide nanoparticles [56], to be used in pharmacy (drug delivery systems) and during production of novel carboxymethyl starch-based biodegradable films with calcium montmorillonite that can be used for the production of seed tapes [55].

Heating a water suspension of starch, glycerol, and citric acid to the temperature of 75 °C and 85 °C was used to produce biodegradable and not-retrogradable eco films [14]. Authors of this work emphasized that, under these conditions, the reaction of esterification proceeds in two stages. In the first stage, citrate esters of glycerol are produced, and in the second stage, these esters react with starch, forming crosslinking bonds.

## 3. Properties of Starch Citrate

The esterification and crosslinking of starch with citric acid causes significant changes in its properties, e.g., in its granule morphology, in its rheological and thermal properties, in its susceptibility to retrogradation and syneresis, in its solubility and swelling power, and in its susceptibility to the action of amylolytic enzymes (Table 2). All of this properties described in the article refer to the features of native starch and their changes under the influence of reagents and parameters of the curse of modification, which are included in the studies cited in the article.

The changes observed on the surface of the esterified starch granules included corrosion and fissures. These changes were noticeable in modified preparations produced with the “wet” method [50,53,54], through roasting (the “dry” method) [37,38,43,44,47,51,60], with the electrochemical method [33], through microwave discharged cold plasma [36], and with the reactive extrusion method [34]. Their intensity was varied, and in extreme cases, it led to changes in starch granularity [33,49,58].

Independently of the changes observed on the surface of granule, internal changes were also observed in the starch structure. No changes in the crystallinity pattern were observed, but changes in the relative crystallinity were observed in the citric acid esterified starch preparations. A decrease in the relative crystallinity was observed in starches of diverse origin (Table 2). The changes involved starches of tuber crops like potato [43,58], sweet potato [43,52], and cassava [43,60] and starch including wheat [37], rice [44], or corn [33]. However, Remya et al. reported an increase in the crystallinity of lentil and banana starches after their modification [43]. These results might be due to a difference in the branching patterns of amylopectins in these starches, which plays a key role in the determination of the type of the unit packing and X-ray diffraction pattern. The relative crystallinity remained unchanged in the preparations produced with the use of cold plasma [38]. Several authors have shown a correlation between the degree of substitution and changes in crystallinity. Hernandez-Jaimes et al. demonstrated a gradual decrease in crystallinity content with an increase in the degree of substitution of corn starch citrates produced by the electrochemical method [33]. A similar relationship was observed by Mei et al., who tested cassava starch esterified with various doses of citric acid [46].

Esterification with citric acid influenced the affinity of modified preparations to water. The swelling power of citrates was mostly lower than that of the native starch from various sources (Table 2) [32,42,51,52,54,58,60]. Remya et al. observed an increase in swelling power for citrate obtained from potato, sweet potato and cassava starch modified by various dose of citric acid [43]. Butt et al. showed that the swelling power of cold-water-soluble rice starch citrates prepared with the alcoholic-alkaline method was higher than that of the native starch [38]. Higher swelling power compared to the native corn starch was also exhibited by the preparations produced using microwave-discharged cold plasma [36]. Lee et al. [58] demonstrated a correlation between swelling power and pH value of the esterification reaction, whereas the work by Kapelko-Żeberska et al. [42] demonstrated a correlation between swelling power and roasting temperature. A number of studies reported that starch preparations esterified with citric acid were determined for their water absorption capacity [38,43,50,53]. The esterification process either increased [38,43,50] or decreased [53] its values, and the different outcome of the process was due to the use of various raw materials and esterification conditions because water absorption is affected by contents of amylose and amylopectin as well as by the pattern and degree of starch crystallinity. Conditions of the esterification reaction and starch type determined the solubility of starch citrate esters. The greatest number of studies demonstrated lower solubility of the esters compared to the native starch [32,42,46,54,60]. In turn, Kim et al. and Xia et al. [36,51] indicated an increase in solubility of starch citrate in comparison to an unmodified starch. Some authors found that the changes in the affinity for water of starch citrates were correlated with the degree of substitution. Kim et al. and Mei et al. observed a decrease in the solubility and swelling power of starch citrate, whereby the greater the changes, the higher the degree of substitution [44,46]. However, Hong et al. demonstrated no similar correlation. They showed that the affinity for water of esterified starch did not depend on the degree of substitution but on the technology process using for modification. Starch, which was primarily substituted by citric acid, had higher solubility and swelling power than cross-linked starch [29].

Esterification/crosslinking with citric acid also affected the swelling power and the solubility of starch nanoparticles, the barrier properties (water vapor permeability and moisture absorption) of starch films and starch foams [12], and the release of substances from hydrogels [57].

Thermal properties of starch esterified with citric acid differed from those of native starch obtained from various botanic sources (Table 2). Several studies demonstrated a decrease of phase transition temperature and a decrease of phase transition heat of the citrates compared to native starch [38,42,44,46,47,49,50,54]. The modified preparations produced via the microwave-discharged cold plasma treatment, having a crystallization degree similar to that of native starch, exhibited similar phase transition temperatures and transition heat to native starch [36]. Hernandez-Jaimes et al. showed a decrease in the gelatinization parameters with the degree of substitution [33]. This observation was also confirmed by Mei et al. Furthermore. They found that gelatinization parameters of the samples of citrate starch could not be determined when the degree of substitution was more than 0.129 [46]. Numerous studies have also reported that, due to the damage of the crystalline structure, preparations with a higher degree of substitution did not show a gelatinization peak [37,42,44,47].

The conditions of the esterification process with citric acid influenced rheological properties of the modified preparations. The majority of research demonstrated a significant decrease in the viscosity of pastes [32,33,38,43,51,53,54]. However, some of the preparations were incapable of paste formation [58,61]. Paste viscosity either increased or decreased depending on reaction conditions [35,36].

The changes in pasting temperature caused by the esterification process varied in comparison of the native starch. Investigations conducted by Kim et al. and Falade and Ayetigbo [44,51] showed that the pasting temperature of the modified preparation was lower than that of the native starch, whereas other authors claimed the opposite [32,34]. In turn, some studies [33,36,54] also showed changes to the pasting temperatures or their increase or decrease, depending on the reaction conditions.

Recent research reported on changes in other properties of starch esterified with citric acid, including foam capacity and foam stability [50], starch paste clarity [38,44,51], freeze–thaw stability [31,33,38], color parameters [50,54], results of oscillatory rheological measurements [39], results of texture profile analysis [32,51], and digestibility [47].

Most of the obtained citrates are characterized by reduced solubility and swelling power and lower viscosity and thermal characteristic compared to the native starch (Table 2). Starches of various botanical sources showed similar change trends. However, previous research reported that the properties of starch citrate depend on the genotype of the raw material. Kim et al. found that high-amylose rise starch was more susceptible to substitution with citric acid than normal rice starch [62]. A similar relationship was observed for corn waxy starch. The citrate showed lower solubility and higher swelling power and gelatinization parameters than normal corn starch [29]. Liu et al. indicated that the debranching waxy corn starch had higher degree of substitution level and resistance to amylolysis and had a decreased relative crystallinity compared to non-debranching esterified starch [63].

Nevertheless, significant influence on functional and thermal properties of starch that occur during esterification with citric acid were caused by changes in the structure of the molecule caused both by the degradation of the glucose chains and the formation of new bonds. Singh et al. demonstrated that the solubility and swelling power of starch is inhibited by cross-linking between starch chains and reactive agents [61]. Starches in which that cross-links are more concentrated have lower affinity to water relative to starches which are primarily substituted by citric acid [29]. Zavareze and Dias concluded that the reduction in swelling power of starch citrate was due to the increased interaction between amylose and amylopectin molecules and strengthened intramolecular bonds [64]. In turn, Hung et al. reported a lower water capacity of starch citrate due to the presence of short chain molecules produced by acid hydrolysis. The partial depolymerization of amylopectin and amylose producing short linear and branched chains from both crystalline and amorphous regions, resulting also in the reduced viscosity of starch modified by citric acid and low gel-forming ability [25]. This decrease in viscosity appeared in most of starch citrate preparations presented in Table 2. The parameters of applied chemical modifications and the properties of the basic starch used to change its characteristics are important for individual changes in the properties of the modifications compared to the native starch.

## 4. Starch Citrates as Resistant Starch

One of the key properties of starch esterified/crosslinked with citric acid is its increased resistance to the action of amylolytic enzymes, i.e., the formation of resistant starch (RS) being a fraction of dietary fiber. This property of starch has been already mentioned in the 1970s in the first studies on starch citrates [7]. Resistant starch is subdivided into five types: RS1, RS2, RS3, RS4, and RS5. Chemically modified starch is classified as type RS4 [65].

The appropriate intake of the soluble and insoluble dietary fiber ensures multiple health benefits both in the prophylaxis and treatment of many diseases [17,66,67,68,69,70,71,72]. Dietary fiber and its resistant starch fraction are nutrients for the symbiotic intestinal flora; they stimulate its growth (e.g., in the case of *Ruminococcus bromii*, bifidobacteria, and lactobacilli) while inhibit the growth of opportunistic or pathogenic microorganisms (e.g., clostrnidium) [66,73,74].

Fermentation of dietary fiber leads to the formation of short chain fatty acids (SCFAs), i.e., butyric acid, propionic acid, valeric acid, isovaleric acid, and hexanoic acid [73,75]. A higher content of SCFAs decreases the pH value of colonic digesta [66,68], enhances mucin synthesis, ensures better tightness of the intestinal barrier [63,71], regulates the composition and numbers of intestinal microflora, reduces the inflammatory conditions (e.g., in kidneys of type 2 diabetic patients) [71], and enhances vitamin D3 synthesis [68,71]. The mentioned properties of fiber have also been confirmed in the case of the citrate that consist of the RS4 type of starch. Sorndech et al. studied the impact of starch citrate on the human microbiota and organic acid production. They found a positive effect of RS on the composition of bifidobacteria and lactobacilli and, at the same time, a decrease in the number of clostridia and bacteroides was noted. RS was fermented to a high amount of butyric acid and propionic acid. The chemically modified starch also showed a high probiotics index [76]. In other research, green banana starch, after treatment with the heat moisture in the presence of citric acid, was used to feed rats. The results showed a higher total fecal short-chain fatty acid content and lower fat accumulation in rats fed chemically modified starch [77]. It was also stated that the generation of glucose from citric-acid-treated starch has drastically decreased, which led to a lowering in the glycemic index [78,79]. In vitro digestibility tests of tapioca and maca starch esterified with citric acid indicated a slow digestion rate in term of the increase RS content [47,80]. Zdybel et al. and Sorndech et al. also reported a high resistance of potato or rice starch citrate to amylolysis [76,81]. Probably, the inhibited contact of digestive enzymes within the glucoside bonds of chemically modified starch appears due to the increased spatial hindrance resulting from the attachment of citric anhydride onto the starch molecule [63].

According to the WHO/FAO, a daily demand of an adult man of dietary fiber is 25 g at minimum [82], whereas noticeable therapeutic benefits are observed at its daily consumption of 40–50 g at minimum [71]. Current dietetic guidelines suggest the increase of the intake of low-processed plant-based food products and others rich in dietary fiber. This has been reflected in the research focused on the increasing content of resistant starch in different plant species, e.g., rice [83], and also those addressing novel modification methods of starch added to food products that aim to increase the content of resistant starch at the expense of easily digestible starch in these products [68,84]. A potential source of resistant starch may be offered by starch citrates due to its technological properties and its being harmlessness to the natural environment.

According to recent research, resistant starch content in starch citrates produced with various methods and from different raw materials ranged from a few percent up to 100%. Its content was determined with the method developed by Englyst et al. [85] and with AACC [18] and AOAC [19]. A slightly different approach was employed in studies by Kapelko-Żeberska et al. [41,42], where starch was determined for its resistance to the action of amyloglucosidase. However, the results achieved with this method were comparable to those obtained with the abovementioned commonly used methods [86]. In some investigations [36,37,48,58], RS content was determined both directly in the preparation and after its heat treatment following methodology provided by Minekus et al. [87].

The content of resistant starch in particular preparations depended on multiple factors including the raw material and the conditions of the reaction, such as temperature, pH, reagent dose, and the interactions between them.

Remya et al. [43] concluded that resistant starch content in the preparations modified with citric acid produced under identical conditions (pH, temperature, time, reagent dose) depended on starch type (cassava, sweet potato, banana, lentil starches). Kim et al. reported the effect of corn starch genotype (waxy corn, normal corn) on RS content [35]. Liu et al. used debranched waxy maize starch to produce a citrate-esterified starch. They concluded that debranching led to a higher degree of esterification compared to non-debranched starch [63]. In turn, no correlation was observed between RS content and starch material type during a citric acid esterification of native and retrograded potato starch [42], and of wheat starch and its granule fractions A and B [26].

The susceptibility of modified starch preparations to the action of enzymes is significantly affected by the conditions of the esterification reaction, e.g., reagent dose, pH, and roasting temperature and time.

Literature data published so far [37,43,45,46,88] corroborate a correlation between the amount of citric acid added, the degree of substitution, and resistant starch content in the modified preparation. However, in some research [45,46,62,81], this correlation occurred at citric acid doses lower than 30 g per 100 g of starch. The decreased RS at high citric acid concentrations is likely due to an excess of citric acid. Large amounts of citric acid would induce further hydrolysis of the glucose chains and the collapse of the starch granules [62].

The increased content of resistant starch in the preparation along with an increased dose of citric acid used in the esterification reaction was also demonstrated in many studies [33,36,42,47,58]. Furthermore, some of the research [34,42,58] pointed to a significant effect of reaction temperature on the resistant starch content. These works did not demonstrate a correlation between the substitution degree and resistant starch content. Nevertheless, the recent research indicated positive dependence of RS contained in starch modified with citric acid and the degree of substitution. Liu et al. obtained citrate-esterified debranched starch with a high degree of esterification and concurrently with high resistance to amylolysis [63]. Similarly, Zdybel et al. reported a lower digestibility of starch preparations with the highest degree of substitution with citric acid [81].

The formation of starch fraction resistance to enzymatic hydrolysis results from both esterification/crosslinking with citric acid and from other modifications of starch under the influence of high temperature and the reaction medium. This thesis is corroborated through results presented in many studies [37,42,48,58], according to which the native starch/without the addition of citric acid/subjected to roasting was characterized by higher resistance to the action of amylolytic enzymes than the non-roasted starch. Independently, the esterification/crosslinking reaction conducted with the same dose of citric acid at various temperatures and various pH values produced preparations differing in RS content [35,42,58]. It was also found that even preparations with a similar degree of substitution produced under various reaction conditions may have substantially different RS contents—from 25 to 100% [42]. Apart from the dose of citric acid, the content of resistant starch produced under conditions of reactive extrusion depended on the extrusion temperature, number of passages, rotational speed of the screw [34], and the addition of galactomannans (guar gum, tara gum, locust bean gum).

## 5. The Application of Starch Citrate in Food Industry

The feasibility of adding starch citrate preparations to food products has already been investigated in the 1990s. Wepner et al. [88] demonstrated that the addition of starch citrates containing ca. 50% RS in the amount of 10% to toast bread, 15% to wafers and pasta, and 20% to extruded products enriched with dietary fibers without significantly compromising their organoleptic traits. These authors suggest that it is feasible to improve the quality of products containing starch citrates through appropriate modifications of the technological process.

Recent investigations have also addressed the effect of the addition of starch citrates as resistant starch preparation on the quality of products [16,32,60].

In turn, Hedagati and Niakousari [32] demonstrated that the addition of starch citrates to wheat flour used for bakery production affected its technological properties. However, these changes may have positive or negative outcomes depending on the product, e.g., slowing down the hardening processes of bakery products through the addition of starch citrate may be useful in products with a desirable soft structure.

It is also feasible to enrich dairy beverages with dietary fiber through the addition of cassava starch citrate. Beverages containing 0.5% of this preparation did not differ in their organoleptic properties from “standard” beverages, whereas these with 1.5% addition of the citrate were acceptable by consumers even though their organoleptic traits were modified [60].

Punia et al. [16] determined properties of pasta with a high addition (10–40%) of barley starch citrate. The results of their study demonstrate that it is feasible to produce pasta containing 20% citrate, which increases the dietary fiber contribution and is, simultaneously, characterized by good organoleptic traits.

Currently, research is being carried out to improve the properties of starch citrates to make them more stable in the technological processes that accompany food production. Kim et al. [62] developed a method of obtaining starch citrate using high-amylose brown rice via retort-type cooking (121 °C, 30 min) with the addition of 30–40 mg/L citric acid and further drying. They obtained a preparation with high RS content at the same time resistant to heat treatment. Therefore, it can be used as packed cooked rice, retorted rice, or frozen rice product. Zehra et al. investigated the impact of citric modification combined with alcoholic-alkaline treatment on selected properties of sorghum and corn starch. This modification conferred heat and shear stability to starches, and the obtained starch citrate preparations may find application in many instant, refrigerated, and frozen food products [7].

## 6. Summary

It may be concluded that the work on starch citrate esters represent a topical and meaningful direction of investigations on modified starch preparations. The possibility of modifying starch preparations by esterification/cross-linking with citric acid allows for the production of modified starch, which can be used in the pharmaceutical, packaging, or food industries. Especially significant may be investigations of resistant starch produced via esterification with citric acid because this method produces preparations exhibiting significant resistance and, simultaneously, good functional properties, which are potentially applicable as health-promoting additives that increase the dietary fiber content of food products.

## Figures and Tables

**Table 1:** Methods of citric starch samples preparation.

Method	Type	Degree of Substitution	References
Dry	Oven	0.01–0.42	[11,35,37,38,39,41,42,43,46,47,52,58]
	Extrusion	0.01–0.03	[31,45]
	Microwave with plasma	0.012–0.015	[36]
Wet	Water suspension	0.002–0.023	[50,51,53,54,57,59]
	Electrochemical	0.02–0.12	[55,56]

**Table 2:** Properties of starch citric preparations.

Starch Source	Parameters Studied	Observed Results	Ref.
waxy maize	acid concentration 0–2.5%, seven days storage in 4 °C 10% concentration of guar gum and tara gum screw compression ratio 1:1, extrussion temperature 70 °C, 85 °C, 140 °C	freeze–thaw stability decrease of digestion	[31]
	acid concentration 1.0 M open circuit potential 1–1.2 V number of 0.5 Hz triangular-shaped potential cycle (50, 100, 200, 400)	reduced crystallinity freeze–thaw stability decrease of digestion	[33]
	acid concentration 10–40% screw compression ratio 1:1, temperature 130–190 °C, twin screw	lower swelling power decrease of viscosity decrease of digestion	[34]
	acid concentration 10–20%, MCP time 20 min, power 900 W oven temperature 135 °C, oven time 1 h,	higher swelling power and solubility increase of viscosity no changes in crystallinity	[36]
wheat	acid concentration 40% temperature of oven 130 °C, time of oven 5 h HMT temperature 100 °C, HMT time 3 h	change the relative crystallinity absent of gelatinization peak	[37]
	acid concentration 0–40%, temperature 140 °C, time 7 h	lower swelling power and solubility decrease of viscosity	[32]
potato	acid concentration 10%, 20%, 40% temperature 100 °C, 130 °C, 160 °C, time 3 h	decrease of solubility decrease in phase transition absent of gelatinization peak	[42]
	acid concentration 20%, 40%, 60% temperature 130 °C, time 2 h	lower swelling power change the relative crystallinity increase water sorption	[43]
	acid concentration 10%, 20%, 30% temperature 150 °C, time 5 h pH = 3.5, 4.5, 5.5	lower swelling power change the relative crystallinity incapable of paste formation	[58]
sweet potato	acid concentration 10–60% temperature 140 °C, time 4 h	lower swelling power change the relative crystallinity decrease enthalpy of gelatinization decrease of viscosity	[52]
rice	acid concentration 1%, 10%, 30% temperature 140 °C, time 4 h	lower swelling power change the relative crystallinity decrease of phase transition absent of gelatinization peak decrease of viscosity clarity of starch paste	[44]
cassava	acid concentration 10–40% temperature 130 °C, time 5 h	lower swelling power and solubility decrease of phase transition absent of gelatinization peak	[46]
	acid concentration 10% 30%, 50% temperature 100 °C, 120 °C, 140 °C, time 5 h	decrease of phase transition absent of gelatinization peak lower digestibility	[47]
	acid concentration 40% temperature 135 °C, time 5 h	lower swelling power and solubility change the relative crystallinity incapable of paste formation	[60]
taro	acid concentration 40% temperature 100 °C, time 0.5 h	decrease of phase transition absent of gelatinization peak decrease of viscosity	[49]
yam	acid concentration 15% water:starch suspension ratio 400:300 (*v*/*w*) temperature 27 ± 2 °C, time 5 h	increase of water sorption decrease in phase transition foam stability	[50]
	acid concentration 15%, water:starch suspension ratio 400:300 (*v*/*w*) temperature 28 ± 2 °C, time 5 h	lower swelling power higher solubility decrease of viscosity clarity of starch paste	[51]
acha and iburu	acid concentration 15%, water:starch suspension ratio 400:300 (*v*/*w*) temperature room (25 °C), time 5 h	increase of water sorption decrease in phase transition temperature decrease of viscosity decrease enthalpy of gelatinization	[53]
kidney bean	acid concentration 15% water:starch suspension ratio 400:300 (*v*/*w*) temperature 27 ± 2 °C, time 5 h	lower swelling power and solubility decrease in phase transition temperature decrease of viscosity	[54]
